# The Effect of comprehensive rehabilitation on Lithuanian adolescent’s nonspecific low back pain, depending on the duration: Nonrandomized single-arm trial

**DOI:** 10.1097/MD.0000000000030940

**Published:** 2022-10-14

**Authors:** Tomas Aukštikalnis, Romualdas Sinkevičius, Odeta Rašimaitė, Aurelija Šidlauskienė, Aurelija Emilija Aukštikalnytė, Audrius Dulskas, Eugenijus Jasiūnas, Juozas Raistenskis

**Affiliations:** a Vilnius University, Faculty of Medicine, Vilnius, Lithuania; b Vilnius university hospital Santaros Clinic, Vilnius, Lithuania; c Academic Teaching Department of Medical University Innsbruck, Bolzano/Bozen, Italy; d National Cancer Institute, Vilnius, Lithuania.

**Keywords:** adolescents, comprehensive rehabilitation program, duration, isokinetic training, low back pain, nonspecific

## Abstract

To determine pain and functional changes during comprehensive rehabilitation (CR) in adolescents with nonspecific low back pain (NLBP), and to determine the optimal CR duration.

**Methods:**

The study included 106 adolescents (39 boys [36.8%], 67 girls [63.2%]), 14 to17 years old, with the following inclusion criteria: duration of NLBP for at least 12 weeks; conservative NLBP treatment was effectless; pain intensity using the visual analogue pain scale (VAS) ≤ 7 points; disrupted daily activities; ability to understand and answer the questions; written consent to participate voluntarily in the study. The pain was assessed using the VAS scale, functional changes were assessed using the Oswestry Disability Index (ODI), 12-Item Short Form Survey, Hospital Anxiety and Depression Scale (HAD), and physical functional capacity and proprioception (Proprio) were assessed using an isokinetic dynamometer. The participants performed a comprehensive pain rehabilitation program consisting of physiotherapy, TENS, magnetotherapy, lumbar massage, and relaxing vibroacoustic therapy. The active CR cycle lasted for 22 sessions (with intermediate measurements after 5 and 16 sessions), after which we performed passive observation for another half a year. Five measurements were performed.

**Results:**

Pain, functional assessment, and physical capacity were improved with CR. Statistically significant improvement became apparent after 5 CR sessions, but statistical and clinical significance became apparent after 16 CR sessions. In the distant period, after the completion of CR, neither statistical nor clinical changes occurred.

**Conclusions:**

CR is effective in reducing pain, and improving functional state and physical capacity quickly and reliably in 16 CR sessions, which is sufficient to obtain clinically satisfactory CR results. Good results were achieved during CR and neither improved nor deteriorated spontaneously in the distant period. This study shows a possible mismatch between NLBP intensity and impaired functional state in adolescents.

## 1. Introduction

A hypodynamic lifestyle and its side effects, including obesity, low level of physical activity, abnormal posture, chronic diseases exacerbations, unhealthy lifestyle, and back pain, have been spreading rapidly.

Factors such as age, gender, professional sports participation,^[1–4]^ prolonged periods of sitting (also at school),^[5]^ school backpack habits^[6]^ or “overweight,”^[7]^ psychological factors,^[8]^ hypodynamic lifestyle may lead to nonspecific low back pain (LBP). LBP occurs in younger and younger people, with a significant increase in LBP among adolescents and children: 48% in 15 years^[9]^ and up to 74.4% in children and adolescents up to 18 years.^[10,11]^ Childhood LBP tends to recur in up to 60.5% of cases and become chronic in up to 11.3%.^[12]^ It is also one of the most important risk factors for LBP in occurring^[9,13–18]^ for adults. LBP in adolescence recurs in 84% of adult cases and is more intense than that in the general population.^[19,20]^ High intensity and high probability of recurrence^[17,21]^ characterize LBP in adolescents LBP. A good pain treatment prognosis is shown if assistance is provided timely.^[17,21]^

As an adolescent’s LBP treatment method, physical therapy is well known as an effective LBP treatment method.^[22–24]^ However, there is a lack of evidence on the effectiveness of multidisciplinary rehabilitation programs, especially regarding the impact of rehabilitation program duration, composition, and intensity. The full comprehensive rehabilitation (CR) program consisted of physical therapy, physical agents, massage manipulation, and psychological intervention (in our case vibroacoustic relaxation).

Our goal was to determine pain and functional changes during CR in adolescents with nonspecific low back pain (NLBP) and to verify the optimal CR duration.

### 1.1. Definitions

*Adolescence* is the phase of life between childhood and adulthood, from ages 10 to 19. Adolescence begins with the onset of physiologically normal puberty, and ends when an adult identity and behavior are accepted.^[25–27]^

LBP is defined as pain localized between the 12th rib and the inferior gluteal folds, with or without leg pain. *nonspecific LBP* is defined as back pain with no known underlying pathology.^[28]^ NLBP is defined as LBP not attributable to a recognizable, known specific pathology (e.g., infection, tumor, osteoporosis, fracture, structural deformity, inflammatory disorder, radicular syndrome, or cauda equina syndrome).^[29]^

CR was defined as systematic multidisciplinary treatment given by physicians and health professionals. Individual assessments and treatment plans targeting defined treatment goals were required. The rehabilitation programes should include physical therapy with exercise aiming at improved aerobic fitness, muscle strength, mobility and balance, occupational therapy, and self-management programes.^[30]^

## 2. Methods

### 2.1. Ethical considerations

Before the CR intervention, each adolescent and their parents were asked to sign a written informed consent. Lithuanian Regional Bioethics Committee approved this study (number 6B-11-95 and update 3-1189-703).

### 2.2. Sample size estimation

To calculate of the approximate sample size, we used G*Power 3.1.9.7 Universität Düsseldorf, Germany. F test family → statistical test—Manova: repeated measures, within-factors → type of power analysis—a priori: compute required sample size—given α, power, and effect size were chosen. We chose α err prob - 0.05, 1–β err prob - 0.95, number of groups - 1, number of measurements—4 (as the last measurement is in the distant period), assumed medium partial eta-squared - 0.06, so the effect size is 0.25 and the total sample size was 72. Before the study, we did not know the data probability to meet the normal distribution, therefore according to statistical basics,^[31]^ we calculated the sample size by normal distribution and added 15% for the non-normal distribution case. Finally, we added 15% to the incomplete comprehensive pain rehabilitation program and the total sample size was at least 96.

### 2.3. Participants

Patients of both sexes, with a history of at least 3 months of nonspecific LBP and meeting the inclusion and exclusion criteria, were recruited to a single-arm trial was conducted at Vilnius University Santaros clinics “Children’s Physical and Rehabilitation medicine outpatient department,” from 2017 to 2021 September.

Inclusion or exclusion of the study was determined during an interview by asking about the history of the specific LBP, performing a physical examination, and performing laboratory tests if needed.

Study inclusion criteria:

1. Age from 14 to 17 years.
2. Patients who have experienced lower back pain for at least 12weeks and who meet the conditions for the definition of NLBP.
3. Conservative (pharmaceutical) LBP treatment was effectless.
4. Pain according to visual analogue pain scale (VAS) ≤ 7 points.
5. Disrupted daily activities.
6. Ability to understand and answer the questions of the tests and questionnaires used in the research.
7. Patients, who voluntarily agreed to participate in the study, as well as with the written consent of the parents.

Study exclusion criteria:

1. Pain duration was less than 12 weeks or more than 12 months.
2. Pain intensity exceeds 7 points according to VAS.
3. Back pain of secondary origin was identified in the initial examination as cauda equina syndrome, progressive motor deficiency, clinical signs of nerve root damage (superficial sensory disturbance in the lumbar nerve innervation zone, leg muscle weakness according to myotomes, knee or Achilles tendon reflex changes), inflammation, infection, oncology or trauma, known metabolic causes (like Hypovitaminosis D, Calcium deficiency, malnutrition).
4. Inability to actively and continuously participate in the established comprehensive pain rehabilitation program, including cognitive disturbances.
5. Incomplete rehabilitation program.
6. Syndrome of increased psycho-emotional lability has been identified.

Study termination criteria:

1. Severe pain exacerbation, VAS > 7.
2. Refusal to participate in the assessment.

### 2.4. Research design

Nonrandomized, controlled, single-arm trial was performed. The assessment of patients was performed 5 times in cycles: the first, at the beginning of the rehabilitation course; the second, after 5 sessions; the third, after 16 sessions; and the fourth, after 22 sessions. The last examination was performed 6 months after ending the rehabilitation program (distant period). Such a measurement plan was chosen, considering the specific rehabilitation plan in Lithuania: the initial outpatient stage of rehabilitation lasted 5 sessions, classical outpatient rehabilitation lasted 16 sessions, and inpatient pain rehabilitation lasted 22 sessions.

### 2.5. Intervention

The patients were covered by a traditional CR program that included the following:

2.1.1. Physiotherapy (5 times a week for 30 minute): Lumbar/core stabilization exercises.
2.1.2. Transcutaneous electrical nerve stimulation (5 times a week for 20 minute): for the reduction of pain and muscle tension.
2.1.3. Magnetotherapy (5 times a week for 20 minute): to improve blood microcirculation and reduce muscle spasms.
2.1.4. Lumbar massage (3 times a week for 30 minute, odd days): to reduce muscle tension and spasms, and improve blood circulation/microcirculation.
2.1.5. Relaxing vibroacoustic therapy (2 times a week for 30 minute, even days): improves the psycho-emotional state, reduces muscle tension, and reduces pain. The procedure was performed on a special chair with a vibroacoustic sound system.^[32]^

### 2.6. Assessment

On the first visit, a routine rehabilitation/clinical assessment was performed (body measurements, pain anamnesis, trunk flexibility, muscle palpation, neurological examination, skin reflexes, etc). In addition to this biomedical research, questionnaires and tests were completed repeatedly and individually during each interview by the research design.

**Figure 1.**
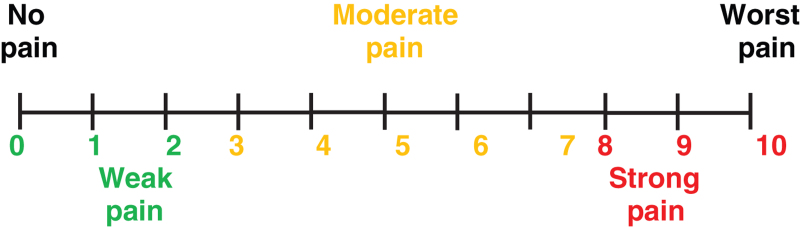
Visual analog pain scale.

2.1.6. *VAS* scores are measured on a 10 cm horizontal line divided into 10 fields, from “0” (no pain at all) to “10” (worst imaginable pain) (Fig. 1).
2.1.7. *Oswestry Disability Index* (ODI)–assesses the influence of lumbar pain on a patient’s functional condition and associated disability.^[33]^ This assessment is used as a “gold standard” tool for LBP in adults.
2.1.8. *SF-12*: 12-Item Short Form Survey assesses patients’ quality of life.^[34]^ It consists of a physical component score (PCS or SF12PCS) and a mental component score (MCS or SF12MCS). We evaluated MCS and PCS separately, as they provide different types of information. We used the online calculator developed by John E. Ware Jr.: https://orthotoolkit.com/sf-12/.
2.1.9. *The Hospital Anxiety and Depression Scale* (HAD) obtains estimates of psycho-emotional status and determines the level of depression and anxiety.^[35,36]^ HAD anxiety component—HADA, HAD depression component—HADD. We evaluated HADA and HADD separately because they provide different types of information.
2.1.10. *Musculoskeletal system functionality* (by muscle work parameters) and proprioception (Proprio) were assessed using an isokinetic dynamometer (“Biodex System 4 Pro™”, NY). Before the main evaluation, the patient practiced several times with 25-50% power to avoid the effect of familiarization errors, as well as a warm-up exercise.
  a) Lumbar muscle isokinetic testing. The patient sat on a special testing seat, the chest and pelvis were fastened with drawstrings. The legs were not fastened to avoid additional trunk flexors or extensors activities. The subjects performed concentric contraction of trunk flexion and extension with maximum effort. The angular speed was 30 °/s for 5 contractions and 120°/s for 20 contractions, with a rest period of 30 second. (see https://m.biodex.com/sites/default/files/830450man_08261clr_revb.pdf, Fig. 1.1, page 5).
  b) The lumbar Proprio test was part of the “Biodex System 4 Pro™” assessment protocol. The subject performs actions in a sitting position with their eyes covered: fixed to test chair patient slowly flexes and extends trunk to predetermined +30° or -30° positions, holds that position for 3 second, returns to a neutral sitting position (~90°), and then tries to repeat the destination angle. The test was performed 3 times, and the difference in the error angles was taken as the average.

### 2.7. Statistical analysis

Statistical analysis was performed using the: R statistical software package V 4.1.2 (© The R Foundation for Statistical Computing), RStudio 2021.09.1 Build 372, © 2009-2021 RStudio, PBC, IBM SPSS Statistics V.27, G*Power V. 3.1.9.7 Universität Düsseldorf, Germany. Interval and ratio variables are described as median and interquartile range. Shapiro–Wilk and Kolmogorov-Smirnov tests were used to check the data for normality. Nominal and ordinal variables were characterized by frequencies and percentages across the corresponding subset of the sample. To assess statistically significant difference within the groups we used Friedman’s test and Wilcoxon’s matched-pairs signed-rank test. To measure the effect size, we used Spearman’s correlation coefficient, Kendall’s concordance coefficient, and the r rank biserial correlation coefficient. Relationships between variables were considered statistically significant when the *P* value was less than.05 (*P* < .05) and a statistical test power of 1-ß was equal to 0.95 (1-ß=0.95).

## 3. Results

A total 106 adolescents participated in the study (39 boys [36.8%], 67 girls [63.2%]), 14 to17 years old. Variable median ± interquartile range: age 15.0 ± 1.0; weight in kilogram 55.9 ± 10.4 (from 38.9 to 74.4); height in cm 170.5 ± 13.0 (from 149 to 195); body mass index in kilogram/cm^2^ 19.22 ± 3.0 (from 13.3 to 24.4); pain duration in weeks 19.0 ± 6.0 (from 12 to 26).

### 3.1. Median changes.

**Figure 2.**
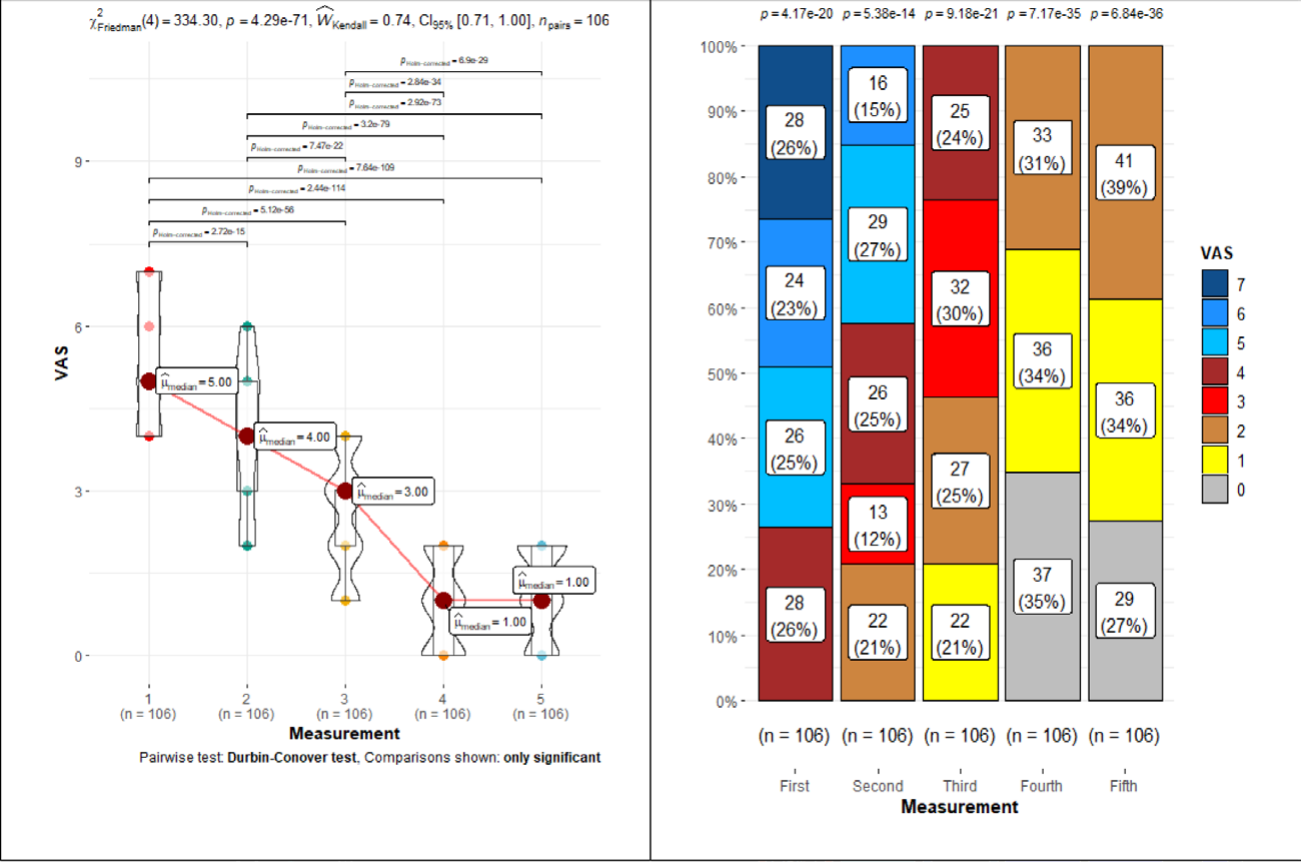
Visual analogue pain scale median and categorical group changes during rehabilitation.

**Figure 3.**
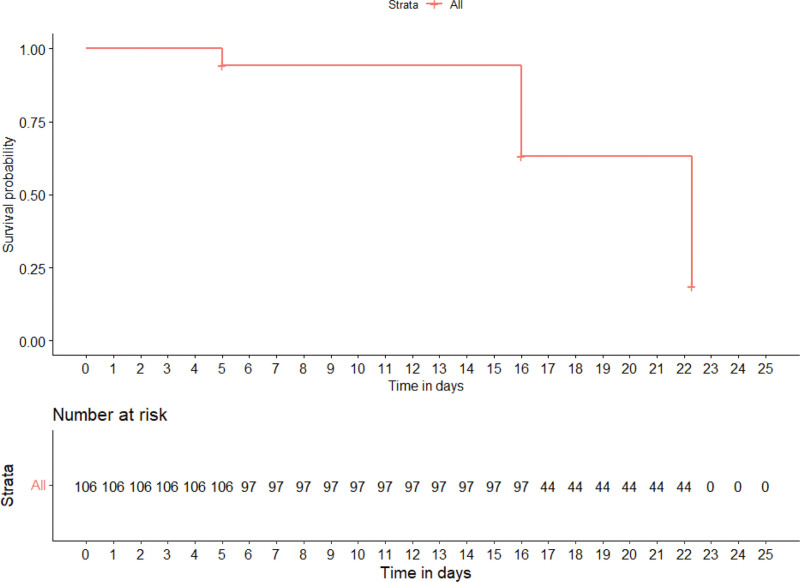
Kaplan-Maier Curve of pain duration time dependence on normalization of patients’ indicators.

VAS showed a clear downward trend during rehabilitation, with a statistically significant difference (*P* < .001): 5→4→3→1→1, except for the last measurement, which showed a statistically insignificant (*P* = .17) difference (Fig. 2 and Table 1). VAS repeated measures Friedman’s test coefficient—334.30, *P* < .001, Kendall’s Coefficient of Concordance—0.74 (large effect). The patients reached a mild pain level (3.0 points) after 16 rehabilitation sessions (Fig. 3). In addition, during rehabilitation, pain categories (determined by pain score) gradually changed, and their number and values decreased statistically significant (*P* < .001) (Fig. 2).

**Table 1:** Variable median changes during rehabilitation.

Meas.	1		2			3			4			5		
Variable	Median	IQR	Median	IQR	**p (1-2**)	Median	IQR	**p (2-3**)	Median	IQR	**p (3-4**)	Median	IQR	**p (4-5**)
VAS	5.0	3.0	4.0	2.0	***	3.0	1.0	***	1.0	2.0	***	1.0	2.0	**ns**
ODI	16.0	4.0	14.0	6.0	***	9.0	4.0	***	2.0	2.0	***	1.0	2.0	**ns**
HADD	4.0	2.0	2.0	2.0	***	1.0	1.0	***	1.0	1.0	***	1.0	1.0	**ns**
HADA	8.0	2.0	3.0	3.0	***	1.0	1.0	***	1.0	1.0	***	1.0	1.0	**ns**
SF12PCS	35.5	11.3	42.1	9.7	***	47.4	7.7	***	52.4	9.0	***	50.7	10.3	**ns**
SF12MCS	42.3	5.4	48.4	14.0	***	56.9	15.4	**	60.8	2.0	***	60.8	3.6	**ns**
Proprioception	6.4	3.8	4.7	2.5	***	1.8	1.8	***	1.45	1.6	**	1.9	1.5	**ns**
EXT 30°/s Peak TQ/BW	147.8	59.2	180.1	81.4	***	239.6	107.3	***	260.5	101.0	***	267.7	104.8	**ns**
EXT 30°/s Total work	351.8	210.8	441.4	262.8	***	574.0	342.2	***	602.2	361.0	***	613.4	365.9	**ns**
FL 30°/s Peak TQ/BW	91.6	30.0	114.0	37.6	***	148.0	50.4	***	155.6	53.0	***	158.8	52.6	**ns**
FL 30°/s Total work	269.3	133.7	336.7	166.3	***	437.6	216.3	***	459.0	225.1	***	468.4	231.1	**ns**
EXT 120°/s Peak TQ/BW	146.3	72.0	182.2	89.3	***	238.5	116.1	***	253.0	120.6	***	256.1	121.1	**ns**
EXT 120°/s Total work	1097.0	903.9	1371.7	1129.6	***	1783.1	1467.1	***	1872.9	1541.0	***	1911.5	1572.6	**ns**
FL 120°/s Peak TQ/BW	100.0	38.8	126.0	47.2	***	163.5	60.3	***	172.0	62.6	***	175.4	64.5	**ns**
FL 120°/s Total work	748.7	675.5	936.3	820.9	***	1217.8	1065.8	***	1278.2	1117.6	***	1304.0	1141.0	**ns**

If *P* value < .001 = ***; .001 to.01 = **; .01 to.05 = *;

HADA = Hospital Anxiety and Depression Scale, depression component score, HADD = Hospital Anxiety and Depression Scale, anxiety component score, IQR = interquartile range, ns = not significant, ODI = Oswestry Disability Index, P = *P* value, SF12PCS = 12-Item Short Form Survey, physical component score, SF12MCS = 12-Item Short Form Survey, a mental component score, VAS = visual analog pain scale score.

Isokinetic assessment: EXT 30°/s Peak TQ/BW = peak torque and bodyweight proportion at 30°/sec speed on trunk extension, EXT 30°/s Total work = total work at 30°/sec speed on trunk extension; FL 30°/s Peak TQ/BW = peak torque and bodyweight proportion at 30°/sec speed on trunk flexion, FL 30°/s Total work = total work at 30°/sec speed on trunk flexion, EXT 120°/s Peak TQ/BW = peak torque and bodyweight proportion at 120°/sec speed on trunk extension, EXT 120°/s Total work = total work at 120°/sec speed on trunk extension, FL 120°/s Peak TQ/BW = peak torque and bodyweight proportion at 120°/sec speed on trunk flexion, FL 120°/s Total work = total work at 120°/sec speed on trunk flexion.

**Figure 4.**
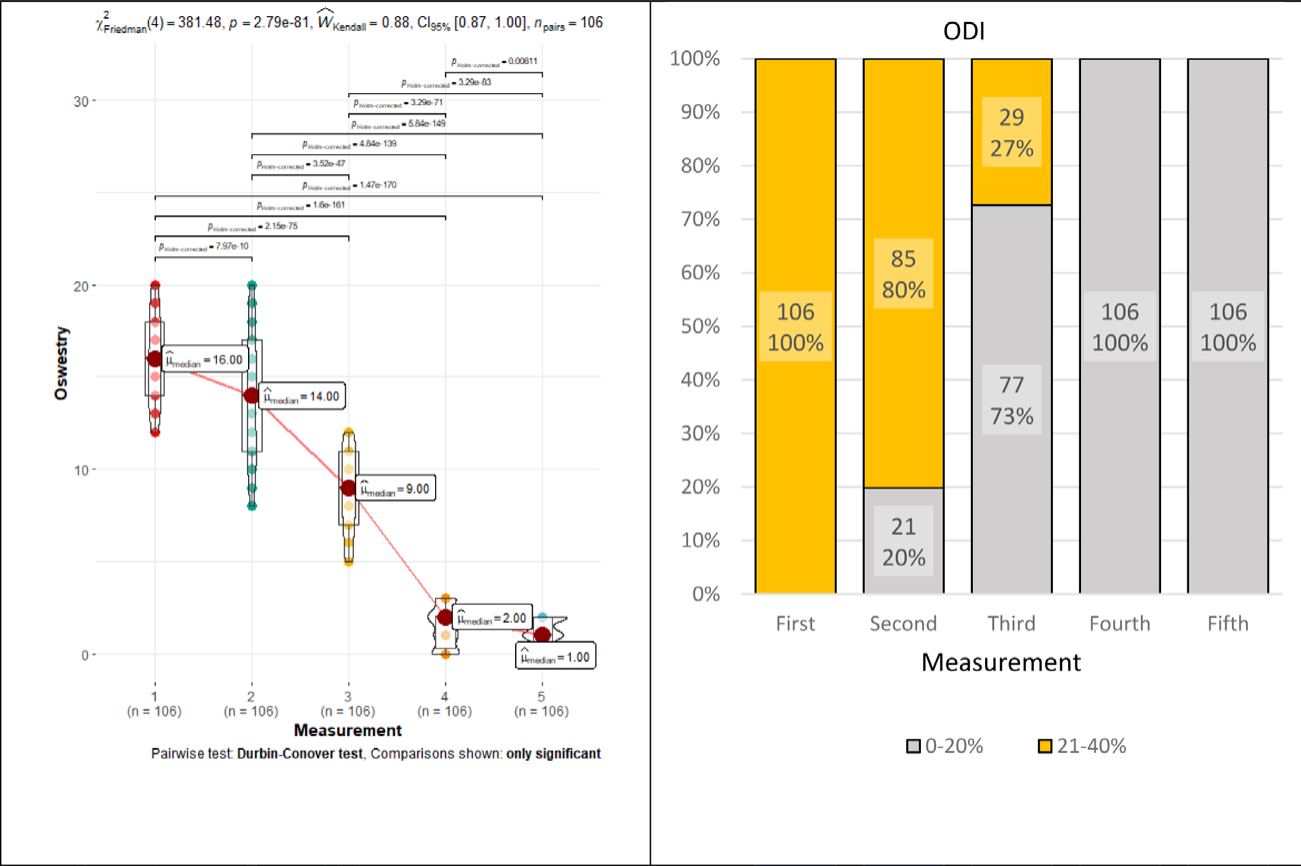
Oswestry Disability Index median and categorical group changes during rehabilitation.

ODI showed a statistically significant downward trend during rehabilitation (*P* < .001): 16→14→9→2→1, except for the last measurement, which showed a statistically insignificant difference (*P* = .15) (Fig. 4 and Table 1). ODI repeated measures Friedman’s tests coefficient—381.48, *P* < .001, Kendall’s Coefficient of Concordance–0.88 (large effect). Patients reached a minimal disability level (<10 points or <20%) after 16 rehabilitation sessions (Fig. 3). The ODI score dramatically decreased after the 22 rehabilitation sessions and became clinically insignificant. In addition, during rehabilitation, ODI categories (determined by ODI score) gradually changed, and their number and values decreased statistically significant (*P* < .001) (Fig. 4).

**Figure 5.**
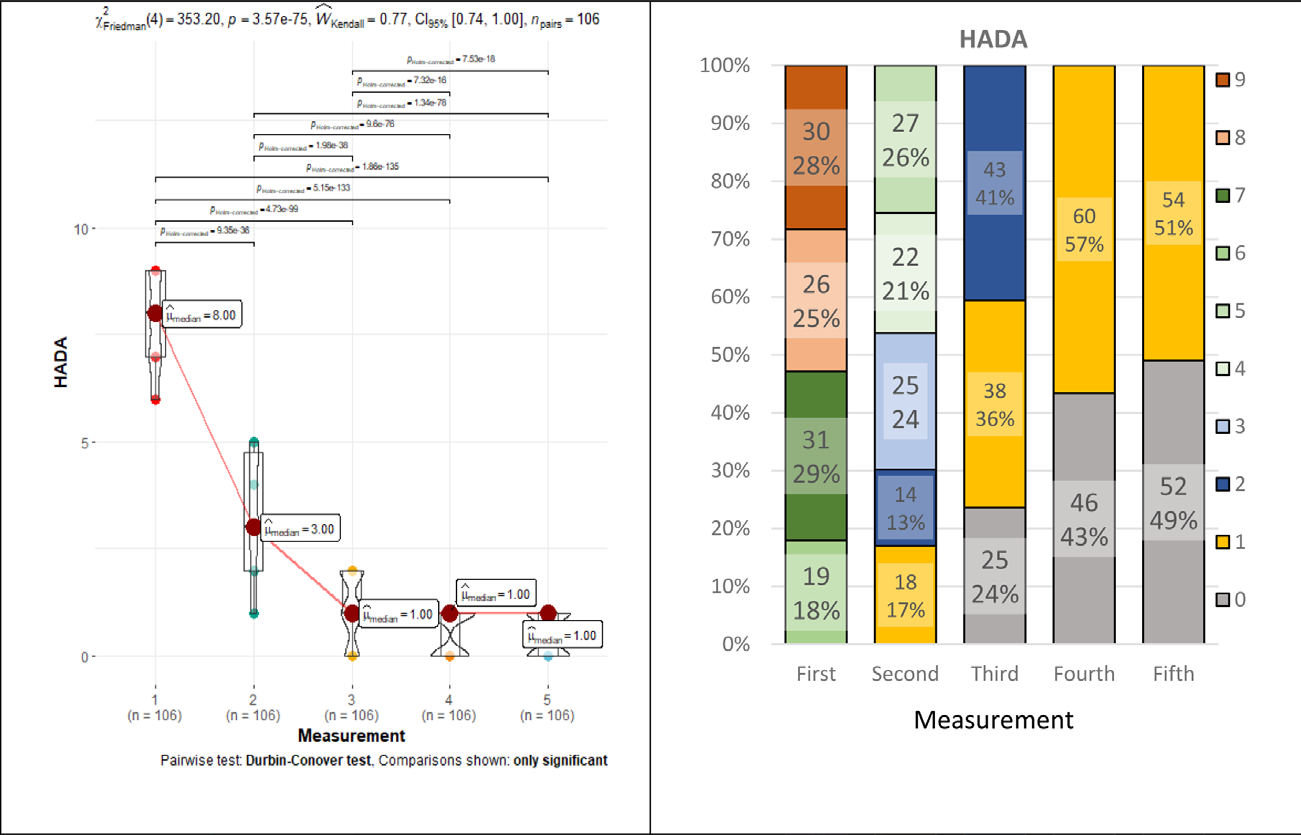
Hospital Anxiety and Depression Scale: anxiety median and categorical group changes during rehabilitation.

The HAD depression score *clinically* was insignificant from the beginning of rehabilitation, with a median of <7 points. Meanwhile, the HAD anxiety (HADA) value at the beginning was assessed as a subclinical symptom level of anxiety (8 points). After the beginning of rehabilitation, anxiety was statistically significant (*P* < .001) and decreases to 3 points, and subclinical symptoms of anxiety were considered clinically insignificant (Fig. 5). Statistically (but not clinically) significant differences remained in the other measurements, except for the last measurement, which was statistically insignificant (*P* = .39) (Fig. 5 and Table 1). The HADA repeated measures Friedman’s test coefficient—353.20, *P* < .001, Kendall’s Coefficient of Concordance—0.77 (a large effect). In addition, during rehabilitation, HADA categories (determined by HADA score) gradually changed, and their number and values decreased statistically significant (*P* < .001) (Fig. 5).

SF-12 assessment results increase all the time during rehabilitation, with a statistically significant difference (*P* < .001), except for the distant period: SF12MCS (*P* = .78) and SF12PCS (*P* = .28).

**Figure 6.**
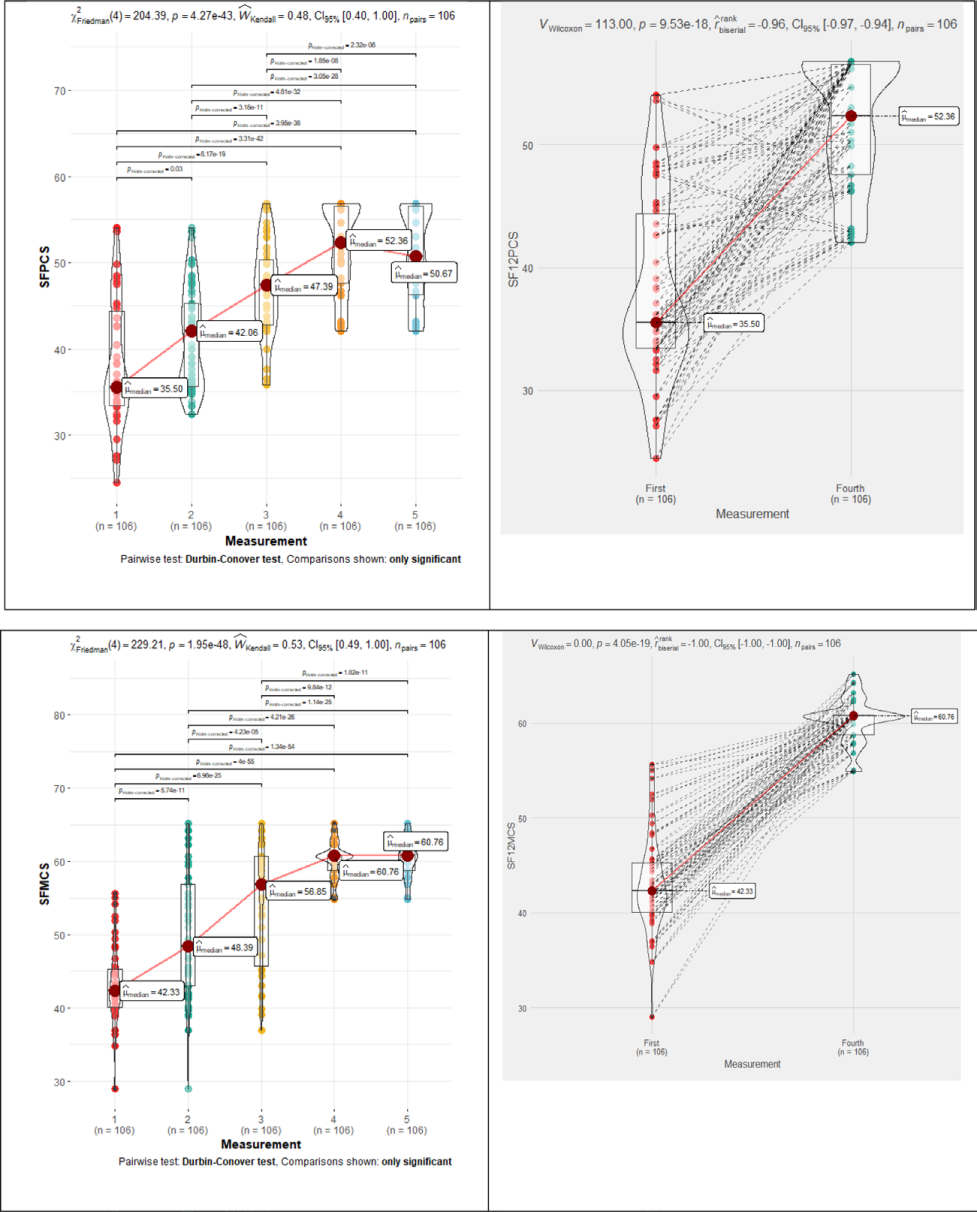
12-Item Short Form Survey physical and mental component median changes during rehabilitation.

a) SF12PCS median changes: 35.5→42.1→47.4→52.4. Repeated measures Friedman’s test coefficient—204.39, *P* < .001, Kendall’s Coefficient of Concordance—0.48 (moderate effect) (Fig. 6 and Table 1). The patients reached the norm (>= 50) after 22 rehabilitation sessions (Fig. 3).
b) SF12MCS median changes: 42.3→48.4→56.9→60.8→60.8. Repeated measures Friedman’s test coefficient—229.21, *P* < .001, Kendall’s Coefficient of Concordance—0.53 (moderate effect) (Fig. 6 and Table 1). The patients reached the norm (>= 50) after the sessions of rehabilitation (Fig. 3).

**Figure 7.**
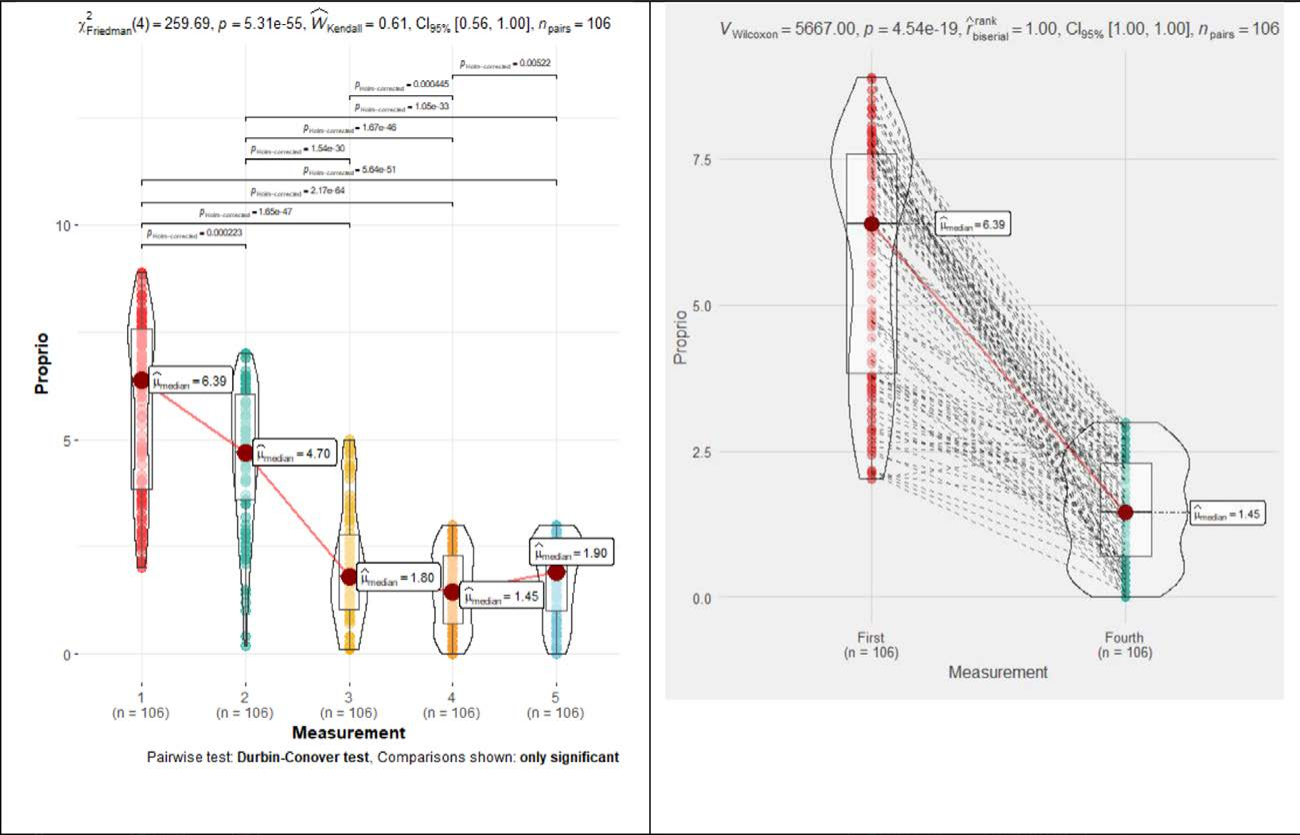
Proprioception median changes during rehabilitation.

The Proprio error in degrees statistically significant (*P* < .001) decreased all the time during rehabilitation: 6.4→4.7→1.8→1.45→1.9, except for the last measurement, which difference was statistically insignificant (*P* = .5). Proprio repeated measures Friedman’s test coefficient—259.69, *P* < .001, Kendall’s Coefficient of Concordance—0.61 (moderate effect) (Fig. 7 and Table 1). If we do not consider the Proprio error of less than 3°, then the patients reached the norm after 16 rehabilitation sessions, and it remains clinically insignificant until the end of the study (Table 1).

Isokinetic assessment: trunk flexion and extension at 30°/s and 120°/s total work and peak torque/body weight results of variables increased all the time during the rehabilitation, showing a positive statistically significant (*P* < .001) result, except for the last measurement, which was statistically insignificant (p from.42 to.78) (Table 1). The repeated-measures coefficients and concordance are shown in Table 2.

**Table 2:** Trunk muscle isokinetic assessment repeated measures indicators.

Variable	Friedman’s tests coefficient	*P* value	Kendall’s coefficient of concordance	Effect
EXT 30°/s Peak TQ/BW	284.974	<.001	0.672	moderate
EXT 30°/s Total work	221.751	<.001	0.523	moderate
FL 30°/s Peak TQ/BW	284.974	<.001	0.674	moderate
FL 30°/s Total work	241.238	<.001	0.569	moderate
EXT 120°/s Peak TQ/BW	257.532	<.001	0.607	moderate
EXT 120°/s Total work	188.438	<.001	0.444	moderate
FL 120°/s Peak TQ/BW	292.928	<.001	0.691	moderate
FL 120°/s Total work	195.645	<.001	0.461	moderate

EXT 30°/s Peak TQ/BW = peak torque and bodyweight proportion at 30°/sec speed on trunk extension, EXT 30°/s Total work = total work at 30°/sec speed on trunk extension, FL 30°/s Peak TQ/BW = peak torque and bodyweight proportion at 30°/sec speed on trunk flexion, FL 30°/s Total work = total work at 30°/sec speed on trunk flexion, EXT 120°/s Peak TQ/BW = peak torque and bodyweight proportion at 120°/sec speed on trunk extension, EXT 120°/s Total work = total work at 120°/sec speed on trunk extension, FL 120°/s Peak TQ/BW = peak torque and bodyweight proportion at 120°/sec speed on trunk flexion, FL 120°/s Total work = total work at 120°/sec speed on trunk flexion;

### 3.2. Correlations

**Figure 8.**
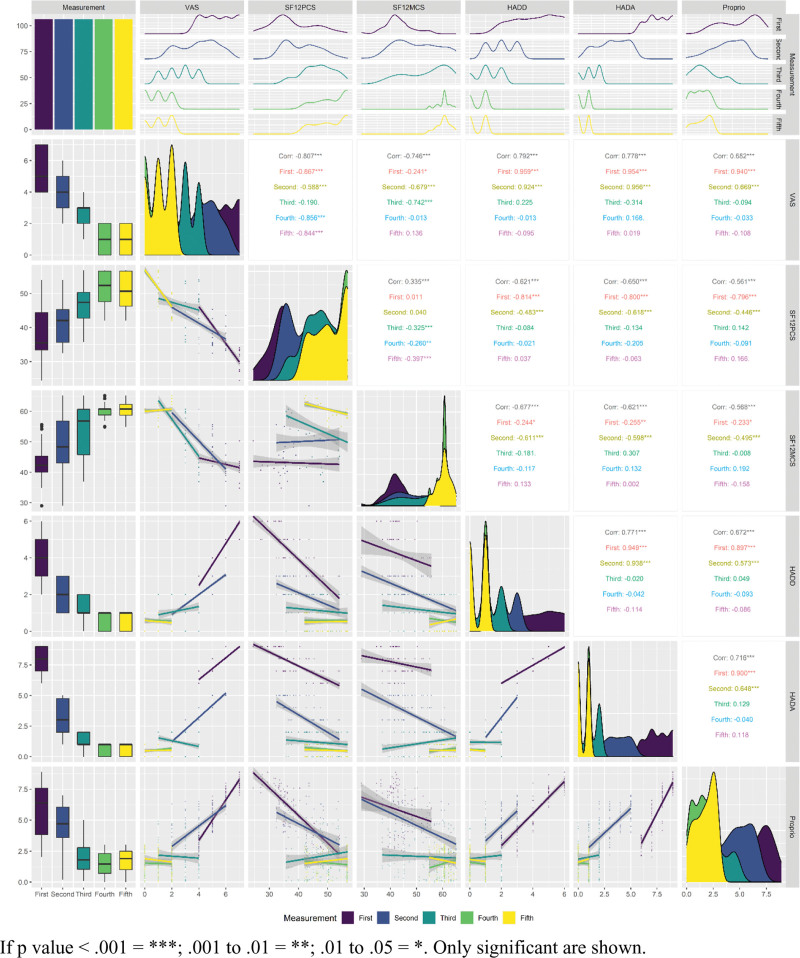
Correlations by measurements: Visual analog pain scale, Hospital Anxiety and Depression components, 12-Item Short Form Survey components.

The statistically significant correlation coefficients are shown in Figure 8 and Table 3.

**Table 3:** Trunk muscle isokinetic variable correlations within the group.

Variable	Meas.	EXT 30°/s Total work	FL 30°/s Peak TQ/BW	FL 30°/s Total work	EXT 120°/s Peak TQ/BW	EXT 120°/s Total work	FL 120°/s Peak TQ/BW	FL 120°/s Total work
EXT 30°/s Peak TQ/BW	1	0.399***	0.413***	0.251**	0.498***	0.275**		
	2	0.509***	0.478***	0.405***	0.506***	0.440***		0.222*
	3	0.315***	0.282**	0.299**	0.301**	0.253**		0.220*
	4	0.515***	0.516***	0.395***	0.575***	0.500***	0.277**	0.277**
	5							
EXT 30°/s Total work	1			0.477***	0.238*	0.273**		
	2		0.282***	0.625***	0.225*	0.462***		0.360***
	3		0.310***	0.324***		0.412***		0.224*
	4		0.272**	0.627***	0.221*	0.463***		0.361***
	5							0.201*
FL 30°/s Peak TQ/BW	1			0.347**	0.313**	0.309**	0.249*	
	2			0.598***	0.544***	0.432***	0.462***	0.472***
	3			0.311***	0.457***	0.310***	0.299**	0.367***
	4			0.599***	0.541***	0.425***	0.477***	0.461***
	5							
FL 30°/s Total work	1					0.305**		
	2				0.383***	0.602***	0.219*	0.664***
	3					0.391***		0.418***
	4				0.382***	0.604***	0.217*	0.665***
	5							0.210*
EXT 120°/s Peak TQ/BW	1					0.210*		
	2					0.636***	0.459***	0.424***
	3					0.551***	0.295**	
	4					0.634***	0.470***	0.425***
	5						0.275**	
EXT 120°/s Total work	1							0.277**
	2						0.307***	0.682***
	3						0.205*	0.397***
	4						0.309***	0.683***
	5							
FL 120°/s Peak TQ/BW	1							
	2							0.443***
	3							0.264**
	4							0.443***
	5							

Only significant are shown. If *P* value < .001 = ***;.001 to.01 = **;.01 to.05 = *.

EXT 30°/s Peak TQ/BW = peak torque and bodyweight proportion at 30°/sec speed on trunk extension, EXT 30°/s Total work = total work at 30°/sec speed on trunk extension, FL 30°/s Peak TQ/BW = peak torque and bodyweight proportion at 30°/sec speed on trunk flexion, FL 30°/s Total work = total work at 30°/sec speed on trunk flexion, EXT 120°/s Peak TQ/BW = peak torque and bodyweight proportion at 120°/sec speed on trunk extension, EXT 120°/s Total work = total work at 120°/sec speed on trunk extension, FL 120°/s Peak TQ/BW = peak torque and bodyweight proportion at 120°/sec speed on trunk flexion, FL 120°/s Total work = total work at 120°/sec speed on trunk flexion.

VAS scores before rehabilitation correlated with HADD (ρ = 0.959, *P* < .001), HADA (ρ = 0.954, *P* < .001), Proprio (ρ = 0.940, *P* < .001), SF12PCS (study group ρ = -0.867, *P* < .001) and SF12MCS (ρ = -0.241, *P* = .013). In the second measurement, VAS scores correlated with HADD (ρ = 0.924, *P* < .001), HADA (ρ = 0.956, *P* < .001), Proprio (ρ = 0.669, *P* < .001), SF12PCS (ρ = -0.588, *P* < .001) and SF12MCS (ρ = -0.679, *P* < .001). In the third measurement, VAS scores correlated with SF12MCS (ρ = -0.742, *P* < .001). At the end of rehabilitation, VAS score correlated with SF12PCS (ρ = -0.856, *P* < .001). In distant period VAS was correlated with SF12PCS (ρ = -0.844, *P* < .001).

HADD scores before rehabilitation correlated with HADA (ρ = 0.949, *P* < .001), Proprio (ρ = 0.897, *P* < .001), SF12PCS (ρ = -0.814, *P* < .001) and SF12MCS (ρ = -0.244, *P* = .012). In the second measurement, HADD scores correlated with HADA (ρ = 0.938, *P* < .001), Proprio (ρ = 0.573, *P* < .001), SF12PCS (ρ = -0.483, *P* < .001) and SF12MCS (ρ = -0.611, *P* < .001). In the third, fourth, and distant period measurements HADD scores were not significantly correlated.

HADA scores before rehabilitation correlated with Proprio (ρ = 0.900, *P* < .001), SF12PCS (ρ = -0.800, *P* < .001) and SF12MCS (ρ = -0.255, *P* = .008). In the second measurement, HADA scores correlated with Proprio (ρ = 0.6483, *P* < .001), SF12PCS (ρ = -0.618, *P* < .001) and SF12MCS (ρ = -0.598, *P* < .001). In the third, fourth, and distant period measurements HADA scores were not significantly correlated.

The SF12PCS scores before rehabilitation correlated with Proprio (ρ = -0.796, *P* < .001). In the second measurement, SF12PCS scores correlated with Proprio (ρ = -0.446, *P* < .001). In the third, fourth, and distant period measurements SF12PCS scores were not significantly correlated.

The SF12MCS scores before rehabilitation correlated with Proprio (ρ = -0.233, *P* = .016). In the second measurement, SF12MCS scores correlated with Proprio (ρ = -0.495, *P* < .001). On the third, fourth, and distant period measurements SF12PCS scores were not significantly correlated.

No statistically significant correlation was established between VAS and ODI, VAS and isokinetic variables, or ODI and isokinetic variables (not shown in figures or tables).

Trunk muscle isokinetic variables were statistically significant correlated within each group (Table 3).

## 4. Discussion

Our study showed that a comprehensive pain rehabilitation program effectively reduced NLBP severity in adolescents, over the course. The optimal duration of CR program for clinical significance was 16 sessions. Good analgesic results, achieved during comprehensive pain rehabilitation, remain for at least half a year. This leads to good pains secondary prevention.

Data analysis confirmed our practical experiences and expectations in dealing with adolescents’ NLBP, as well as the results of other similar studies.^[24,37,38]^ High quality studies are still in a small number (especially not older than 5 or even 10 years); therefore, the comparison is particularly limited.

Some countries have different rehabilitation systems, in which pain monotherapy is used (usually physiotherapy). Therefore, there is a need to quantify the effects of such therapies, depending on their duration, and to compare them with comprehensive pain rehabilitation results.

We found that the ODI was not correlated with pain and other functional assessments or trunk muscle physical capabilities. This describes the ODI as a poor predictor in the case of adolescents. Moreover, pain did not correlate with the physical capabilities of the trunk muscle. This probably means, that despite the decreased in pain intensity, functional disability decreased, and at the same time physical capabilities increased the interconnection was disputable. Further research is needed to explain this phenomenon in adolescents, with special attention paid to the psycho-emotional influence on pain sensation and recognition.

The results achieved at the end of rehabilitation did not change during the distant rehabilitation period. This finding may have been influenced by several factors: 1. Optimal painless body functionality was achieved, and patients improved and no longer needed continuous professional rehabilitation care. 2. The rehabilitation program was continued at home by performing rehabilitation tasks and maintaining a good functional state, which prevented recurrence of pain, but without any spontaneous functional improvement. 3. Medical and rehabilitation care reduces the impact of psycho-emotional status on pain and prevents the pain catastrophizing. 4. The evaluation of the patients did not take into account more potential effects that may impact the results. Further research is needed.

During CR, alternative agents may be used, whose anti-pain and additional effects can be positive, just like reflexotherapy,^[39]^ acupuncture,^[40–42]^ stretching, spinal manipulation,^[18]^ art therapy and so on, witch results depends on usage technique and experience. This was not our subject in this study, but more research is needed to assess the potential positive effects of these instruments, especially if we consider the effects of adolescent psycho-emotional lability on pain perception.

Our study has some limitations. First, during puberty, many biological, neurohumoral, and psychological changes occur quickly in the body. Some sensations are disproportionately overvalued, psycho-emotional management is temporarily impaired, and adolescents are highly dependent on well-being state and mood. This psycho-emotional lability can affect self-estimation and greatly distort self-report answers in a short period and can also lead to pain chronization over catastrophizing.

Second, due to the limitation of height, maximum effort during the isokinetic testing was limited by age, and only 14 to 17 years of age patients were selected. Hence, these results cannot represent the entire population of children with LBP. This was also highlighted by the unequal number of participants of both sexes. The dominance of girls in this study is consistent with the results of LBP in adolescent studies by many researchers. Trying to split groups by sex, due to the relatively small sample size, the study loses its power and provides potentially unreliable results. Because of the Lithuania rehabilitation system, which determined the structure of this research, we obtained asymmetric time intervals between measurements, which may lead to difficulties in comparing the results with those of other similar studies.

## 5. Conclusion

Comprehensive pain rehabilitation, targeted at adolescents’ NLBP, provided by a professional rehabilitation specialist, is effective. A comprehensive pain rehabilitation program reduces adolescents’ NLBP and improves functional state fast and effectively in a short period. 16 comprehensive pain rehabilitation sessions are sufficient to obtain clinically satisfactory pain rehabilitation results. Good results, reached during comprehensive pain rehabilitation, did not change in the distant period: there was neither spontaneous improvement nor continuing deterioration.

This study shows a possible mismatch between adolescents’ NLBP intensity and impaired functional state, due to a lack of correlations.

## Acknowledgments

The authors are grateful to all participants of this study and thankful to the staff of the Children’s Physical and Rehabilitation Medicine Unit of Vilnius University Santaros clinics for providing the prescribed therapy and valuable support or advice.

## Author contributions

**Conceptualization:** Tomas Aukštikalnis, Audrius Dulskas, Juozas Raistenskis.

**Data curation:** Tomas Aukštikalnis, Aurelija Emilija Aukštikalnytė.

**Formal analysis:** Tomas Aukštikalnis, Eugenijus Jasiūnas.

**Investigation:** Tomas Aukštikalnis, Odeta Rašimaitė.

**Methodology:** Tomas Aukštikalnis, Romualdas Sinkevičius, Odeta Rašimaitė, Audrius Dulskas, Juozas Raistenskis.

**Project administration:** Tomas Aukštikalnis, Aurelija Šidlauskienė, Aurelija Emilija Aukštikalnytė, Audrius Dulskas, Juozas Raistenskis.

**Resources:** Tomas Aukštikalnis, Romualdas Sinkevičius, Aurelija Šidlauskienė, Eugenijus Jasiūnas.

**Software:** Eugenijus Jasiūnas.

**Supervision:** Tomas Aukštikalnis, Juozas Raistenskis.

**Validation:** Tomas Aukštikalnis, Romualdas Sinkevičius, Odeta Rašimaitė, Aurelija Šidlauskienė, Juozas Raistenskis.

**Visualization:** Tomas Aukštikalnis, Odeta Rašimaitė, Aurelija Emilija Aukštikalnytė, Eugenijus Jasiūnas.

**Writing – original draft:** Tomas Aukštikalnis.

**Writing – review & editing:** Tomas Aukštikalnis, Romualdas Sinkevičius, Odeta Rašimaitė, Aurelija Šidlauskienė, Aurelija Emilija Aukštikalnytė, Audrius Dulskas.
